# Static vs. Immersive: A Neuromarketing Exploratory Study of Augmented Reality on Packaging Labels

**DOI:** 10.3390/bs15091241

**Published:** 2025-09-11

**Authors:** Sebastiano Accardi, Carmelo Campo, Marco Bilucaglia, Margherita Zito, Margherita Caccamo, Vincenzo Russo

**Affiliations:** 1 Department of Business, Law, Economics and Consumer Behaviour “Carlo A. Ricciardi”, Università IULM, 20143 Milan, Italy; sebastiano.accardi@studenti.iulm.it (S.A.); marco.bilucaglia@studenti.iulm.it (M.B.); margherita.zito@iulm.it (M.Z.); vincenzo.russo@iulm.it (V.R.); 2 Behavior and Brain Lab IULM—Neuromarketing Research Center, Università IULM, 20143 Milan, Italy; 3 Consorzio per la Ricerca nel Settore della Filiera Lattiero—Casearia e dell’Agroalimentare (CoRFiLaC), 97100 Ragusa, Italy; caccamo@corfilac.it

**Keywords:** neuromarketing, consumer neuroscience, augmented reality, emotions, EEG, immersive, packaging

## Abstract

Augmented Reality (AR) is a technology adopted by brands to innovate packaging and improve communication with consumers. Companies integrate AR features into their packaging, choosing between different approaches. However, it is still unclear how different AR typologies can influence consumers’ perceptions during the interaction. For this purpose, this exploratory study aims to analyze the differences between two types of AR—static vs. immersive—applied to packaging, evaluating their impact and effectiveness on consumers. A within-subjects design, on a sample of 20 participants, was employed using neuroscientific techniques (electroencephalography, heart rate, and skin conductance) to explore the cognitive and emotional engagement based on the AR interaction, as well as self-report measures (Augmented Reality Immersion, Perceived Informativeness and Authenticity). Neurophysiological findings indicated that the immersive AR application elicited a greater emotional and partially cognitive engagement, as well as a higher perceived immersion, according to self-reports. The study’s findings offer a deeper understanding of how consumers’ perceptions can change in response to different types of AR content. Although AR is not yet widely accessible as a marketing tool for brands, its growing technological feasibility makes it relevant to know its potential effects on consumers. Thus, this study will offer useful insights for companies to direct their investments toward AR applications in marketing campaigns.

## 1. Introduction

### 1.1. The Application of Augmented Reality in Marketing and Packaging

Among the emerging technologies, Augmented Reality (AR) has attracted increasing attention due to its potential to enrich the individual’s sensory and perceptual experience. AR can accomplish this by overlaying computer-generated elements onto the physical environment through compatible devices (Archana & Stephen, 2025). Thus, AR represents a transformative interface between digital enhancement and human perception, altering how sensory information is processed at the cognitive and neural levels (Archana & Stephen, 2025; Abrash, 2021). Despite this, traditional AR systems often rely on a complex combination of multiple hardware and software components, requiring specialized equipment or a location. In contrast, a simpler form of AR is mobile Augmented Reality, which significantly lowers the barrier to entry by leveraging the built-in sensors and cameras of mobile devices, such as smartphones and tablets (Cao et al., 2023). This offers intuitive and accessible AR experiences for everyday use, often triggered by Quick Response (QR) codes or visual markers (anchors) placed on physical objects (Farshid et al., 2018; Kim et al., 2019). The widespread diffusion of mobile devices, combined with the ongoing miniaturization and decreasing costs of mobile computing platforms, ubiquitous internet access, and advances in mobile cloud computing, has fueled the growth of the mobile AR market and its broader dissemination (Chatzopoulos et al., 2017; Cao et al., 2023), whose value exceeds USD 700 million (Chatzopoulos et al., 2017). Recent Human–Computer Interaction research further extends this view, positioning Extended Reality (XR)—which comprises AR—as the next general computing platform that will dominate our relationship with the digital world for the coming decades (Abrash, 2021). In this perspective, AR solutions are not merely transitional technologies but are central to a broader paradigm shift toward always-available, contextually personalized interfaces that augment human perception and cognition. Given its broad versatility, this highly practical typology of AR finds uses in multiple contexts, such as education (Antonioli et al., 2014; Diaz et al., 2015; Specht et al., 2011; Sungkur et al., 2016), medicine (Barsom et al., 2016), and marketing (Kim et al., 2019; Irshad & Awang, 2016; Scholz & Smith, 2016).

In the marketing field, AR has shown strong potential, emerging as a powerful tool to advertise products (Kim et al., 2019; Irshad & Awang, 2016; Scholz & Smith, 2016). Indeed, major brands such as IBM Corporation, Starbucks, and Volkswagen have already implemented mobile AR solutions in their communication strategies (Adhani & Rambli, 2012). In particular, mobile AR is becoming frequently used in the food and beverage retail industry, where AR apps are often developed through QR codes or image recognition technology embedded in product packaging (Kim et al., 2019; Farshid et al., 2018; Penco et al., 2021; Juan et al., 2019). Indeed, among the AR applications in marketing, product packaging and labels have emerged as strategic touchpoints. Their physical limitations and the direct interface with consumers make them particularly suitable for AR integration, enabling brands to enrich communication, provide additional layers of information, and enhance the overall user experience (Penco et al., 2021; Juan et al., 2019). From a consumer psychology perspective, packaging is not merely an aesthetic container but a strategic communication tool capable of conveying value, particularly when integrated with technologies that enhance multisensory experiences (Petit et al., 2018; Silayoi & Speece, 2007). Mobile AR, therefore, permits brands to overcome the spatial constraints of traditional packaging and, in turn, to provide additional key information about the product, such as nutritional information or traceability but also storytelling or entertainment (Penco et al., 2021; Juan et al., 2019). For example, the AR implementation on 19 Crimes wines allows consumers to enjoy interactive labels depicting characters and telling stories (Farshid et al., 2018). This demonstrates how mobile AR can transform packaging into a useful interactive channel for both the brand and the consumers. However, despite the growing use of AR in packaging, little is known about how different types of AR content specifically affect consumer responses in this context.

### 1.2. Static vs. Immersive

According to previous research, AR implementation can vary based on how content appears during user interactions (Diaz et al., 2015; Specht et al., 2011; Chatzopoulos et al., 2017; Sonnenberg et al., 2025; Javornik et al., 2019; Georgiou & Kyza, 2017; Nhan et al., 2022). Despite no specific distinction between typologies and no clear definition for them arising from the existing literature, the emerging patterns suggest some differences. Especially in cultural and educational contexts, AR applications on mobile devices generally utilize two categories of content: static and dynamic (Diaz et al., 2015; Specht et al., 2011; Chatzopoulos et al., 2017; Sonnenberg et al., 2025). Static content comprises graphical or textual elements that act as stable overlays and do not react to user input, so its appearance remains unchanged during user interaction. This type of content is typically passively consumed rather than actively explored and is primarily used to provide contextual information or supplemental data. For example, static AR apps have been employed in marketing and cultural heritage contexts to overlay fixed informative text about real-world objects (Abao et al., 2018; Javornik et al., 2019), as well as to overlay fixed images that depict virtual objects (Javornik et al., 2019). Notably, applications combining static text and image content have improved user attention and sense of flow during interaction (Javornik et al., 2019). In contrast, dynamic content is characterized by its ability to change over time during the experience. It can include interactive elements such as animations, videos, or 3D objects that evolve in real time, often responding to users’ actions (e.g., movement, tapping, orientation), creating a continuous and responsive motion flow. In educational contexts, AR apps using both static and dynamic content seem to be effective for learning (Diaz et al., 2015; Specht et al., 2011; Sungkur et al., 2016), although dynamic content is perceived as more supportive (Diaz et al., 2015). Scholz and Smith (2016) point out that even in marketing contexts, the most effective augmented experiences are those that offer content manipulation or interaction, as opposed to mere passive viewing.

Beyond this distinction, recent advancements in more dynamic AR have led to the development of increasingly sophisticated user experiences, introducing a further layer of complexity: the degree of immersion (Georgiou & Kyza, 2017; Nhan et al., 2022). Immersiveness in AR can be divided into two complementary and interacting dimensions: technological and psychological (Fan et al., 2022). From a technological perspective, immersion is defined as how computer displays can deliver an inclusive, extensive, surrounding, and vivid illusion of reality to the user’s senses. In this light, immersion refers to quantifiable characteristics (such as multimodal sensory cues or 360-degree spatial tracking) (Fan et al., 2022). From a psychological perspective, immersion is a form of cognitive and emotional absorption (Georgiou & Kyza, 2017), caused by multiple factors, such as interactivity or narrative-driven elements, that actively engage and engross the users (Georgiou & Kyza, 2017; Nhan et al., 2022) and positively influence their enjoyment (Raman et al., 2025). According to the Flow Theory (Nakamura & Csikszentmihalyi, 2009), immersive experiences are more likely to facilitate a deep state of concentration and enjoyment, often referred to as “flow,” which is associated with heightened cognitive processing and positive affect. Recent neuroscientific research supports this claim, showing that the degree of immersion is positively associated with increased neural activity in specific brain areas, such as those related to attention (Xu & Sui, 2021) and working memory (Souza & Naves, 2021). For example, immersive AR has been shown to enhance cognitive processing and engagement at a neural level (Sung et al., 2023). This effect may be explained by its combination of multimodal elements—such as images, audio, and interactive features (Mayer, 2005; Farida & Clark, 2024)—which stimulate richer information processing. Moreover, the Multisensory Integration Theory (Stein & Stanford, 2008) further supports this explanation, suggesting that the simultaneous stimulation of multiple sensory channels (e.g., vision, hearing, touch) can lead to optimized perception and stronger neural processing. In summary, while these theories provide useful frameworks to interpret AR experiences, prior studies lack a systematic categorization of AR content, leaving the distinction between static and immersive forms only partially explored. Yet, prior research has not systematically compared static versus immersive AR content in consumer settings, leaving unclear which approach is more effective.

### 1.3. AR Effects on Consumer Dimensions

Previous research suggests that, in marketing contexts, multisensory experiences can reinforce brand distinctiveness and value in the consumer’s mind (Hultén, 2011), while attributes like technological integration on packaging significantly affect purchase decisions (Silayoi & Speece, 2007). In line with this, mobile AR has been shown to have a positive impact on consumers (Sung, 2021; Micheletto et al., 2025; Irshad & Awang, 2016), improving brand perception (Rauschnabel et al., 2019), informativeness, and positive affective response towards the experience (Qin et al., 2021; Wu et al., 2022; Pozharliev et al., 2022), but also driving purchase intentions through its degree of immersion and interactivity (Raman et al., 2025). As a result, the integration of AR apps into retail environments has been linked to increased sales, particularly for lesser-known brands and higher-priced items (Tan et al., 2022). Therefore, AR appears to be particularly effective in situations characterized by consumer uncertainty, highlighting how this technology can boost product understanding, consumer confidence (Tan et al., 2022), and perceived product authenticity (Penco et al., 2021). Authenticity, understood in marketing contexts as the perception that a product or brand is genuine and conveys a sense of credibility and integrity, plays a crucial role in shaping consumer responses, as it fosters trust and positive attitudes toward the brand, which in turn can increase the likelihood of purchase intentions (Rosado-Pinto & Loureiro, 2024). Mobile AR applications can also strengthen the consumer–brand relationship by extending brand interactions beyond the point of sale and into consumers’ everyday environments. Their accessibility from personal spaces, such as the home, allows brands to establish a more intimate and continuous presence in consumers’ lives (Scholz & Duffy, 2018). Moreover, compared to traditional web interfaces, AR experiences in e-commerce have shown to enhance perceptions of novelty, immersion, enjoyment, and usefulness—leading to positive attitudes and purchase intentions—with immersion mediating the effects of interactivity and vividness on enjoyment and usefulness (Yim et al., 2017). These findings seem to agree with the Media Richness Theory (Tseng & Wei, 2020), according to which ads on mobile devices with higher media richness are more effective at guiding consumer perception and decision-making. Even in AR contexts, the media richness has been shown to influence consumers’ decision-making, willingness to buy, and brand engagement, as well as to amplify cognitive and emotional reactions (de Amorim et al., 2022). Indeed, immersive AR has proven effective in enhancing the emotional impact and perceived value of the experience, and behavioral responses such as continued application use or the intention to acquire the virtually presented products (Nhan et al., 2022). In this regard, the Elaboration Likelihood Model (ELM) (Petty & Cacioppo, 1986) highlights how persuasive messages can be processed through different routes of elaboration, ranging from information processing through cognitive effort to more affective pathways. Through these mechanisms, the type of AR content may indirectly shape key consumer outcomes such as engagement, perceived authenticity, or purchase intention. Beyond conveying information, AR also creates an enjoyable experience for the consumer, offering both hedonic and informative value and effectively combining entertainment with utility (Hagtvedt & Chandukala, 2023; Voicu et al., 2023; Qin et al., 2021; Tabaeeian et al., 2024).

In line with these benefits, AR experiences have been shown to promote consumers’ cognitive engagement, as measured by an increase in alpha (linked to cognitive processing) and theta (linked to working memory) brainwave activity during the interaction with the AR content (Sung et al., 2023). This finding demonstrates how neuroscientific measures could serve as a valid and objective indicator of AR’s effectiveness (Russo et al., 2022b). Although most research on AR content relies on self-report measures, which are susceptible to biases like social desirability and limited introspection, neuroscientific methods offer a more direct and ecologically valid way to study users’ real-time, implicit, and often unconscious reactions to AR experiences (Missaglia et al., 2017; Krugliak & Clarke, 2022; Russo et al., 2022b). Some neuroscientific tools include electroencephalography (EEG), which enables the investigation of cognitive processes mediated by the cerebral cortex—such as attention, cognitive engagement, or cognitive load—along with heart rate (HR) and skin conductance (SC), which provide measures of emotional arousal (Alvino et al., 2020). Several studies have already used them in marketing contexts to analyze the cognitive and affective neurophysiological responses towards mobile AR (Sung et al., 2023; Wu et al., 2022; Pozharliev et al., 2022) or packaging (Liao et al., 2015). For example, mobile AR-based shopping experiences have been associated with reduced cognitive load, as measured by EEG, compared to traditional web-based interfaces (Wu et al., 2022). Additionally, they have been linked to higher physiological arousal, as measured by skin conductance response, compared to traditional ads (Pozharliev et al., 2022). Notably, Pozharliev et al. (2022) also highlighted a dissociation between physiological and self-reported responses, showing that AR ads elicited greater physiological arousal (e.g., skin conductance) than traditional ads at a level despite similar self-reported ratings. This dissociation supports the need to adopt multimodal methodologies that combine implicit (neurophysiological) and explicit (self-reported) data to better understand consumer experiences in AR research, in line with the consumer neuroscience perspective (Hubert & Kenning, 2008). Indeed, most studies rely on self-reported measures, overlooking the potential of combining explicit and neurophysiological data to capture real-time consumer reactions.

### 1.4. Research Gap

Despite the growing body of research exploring the benefits of AR in marketing contexts, little is known about how different types of AR content, particularly in terms of static vs. dynamic elements and levels of immersion, differentially impact consumers. While previous studies have shown that AR can enhance brand and product perception, authenticity, and purchase intentions, they have not systematically compared different types of AR content. Compounding this issue, the existing literature still lacks a clear and consistent categorization or definition of AR typologies, making it difficult to build a coherent framework for evaluating their relative effectiveness. This leaves unclear which approach is more effective in marketing and especially in consumer-facing applications such as packaging, which is a strategic brand touchpoint where AR can overcome physical constraints and enrich consumer experiences through additional elements. Given the wide variety of AR typologies and the widespread investment and implementation of AR in various sectors by brands, especially the food industry, it is essential to determine which type of AR content is most useful to present to consumers. Furthermore, although consumer responses to AR have often been studied through self-report measures, this approach alone may not capture the full spectrum of cognitive and emotional reactions, as these are frequently implicit and unconscious. Indeed, the combination of self-report and neuroscientific measures (e.g., EEG, HR, SC) remains underutilized in this context, since only a limited number of studies have employed multimodal methodologies that integrate both techniques, despite their potential to provide a richer and more objective understanding of AR’s effects. Addressing this gap is crucial to identifying which AR content characteristics most effectively drive consumers’ responses, thereby offering both theoretical contributions to AR research and practical insights for marketers and brand managers.

### 1.5. Hypotheses Development

Given the increasing integration of AR in the food sector and the limited understanding of how different types of AR content affect consumer experience, this study aims to explore how different types of AR content—specifically static versus immersive formats—implemented on food packaging influence consumers’ cognitive, emotional, and behavioral responses. By adopting a multimodal methodology that combines neuroscientific measures and self-report data, the research aims to address the following questions:

**RQ1**: From a neurophysiological perspective, does the immersive AR on the packaging label of a product engage consumers differently compared to the static AR?

**H1a.**  *Immersive AR on packaging label generates more emotional engagement compared to static AR*.

**H1b.**  *Immersive AR on packaging label generates more cognitive engagement compared to static AR*.

The use of neuroscientific tools enables highly precise recording and analysis of user interactions with AR content. This approach allows researchers to go beyond post-experience self-reports, capturing real-time responses as the interaction unfolds. To address the present research question, we focus on two key neurophysiological measures, which are detailed in the following section. The first is emotional engagement, which helps evaluate the degree of affective involvement during the experience. The second is cognitive engagement, which allows us to assess the level of cognitive processing elicited by different types of AR content. Together, these measures offer a more comprehensive understanding of whether AR content developed for marketing purposes triggers distinct and effective patterns of psychophysiological activation.

**RQ2**: From a declarative perspective, does the immersive AR on the packaging label of a product change how the product is perceived compared to static AR?

**H2a.**  *Immersive AR on packaging label generates more Perceived Informativeness (PI) compared to static AR*.

**H2b.**  *Immersive AR on packaging label generates more Perceived Brand Authenticity (PBA) compared to static AR*.

**H2c.**  *Immersive AR on packaging label generates more Perceived Product Authenticity (PPA) compared to static AR*.

**H2d.**  *Immersive AR on packaging label generates more Intention to Buy (ITB) compared to static AR*.

To fully understand how different AR content types work, it is also necessary to consider the rational and behavioral dimensions, which are essential to exploring how AR applications, when tied to a brand or product, can impact the effectiveness of marketing strategies. For this reason, in order to address the present research question, we evaluated how the subjective perception of specific dimensions related to the product featured (such as intention to buy or perceived product authenticity) may be influenced by the AR experience.

**Figure 1:**
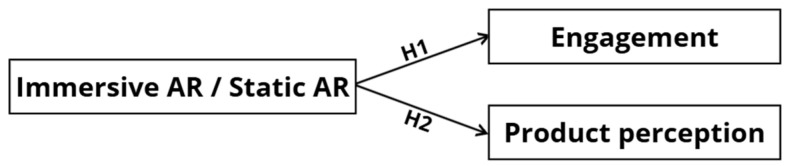
Conceptual model of the study. The AR typology, distinguishing between Immersive AR and Static AR, is supposed to influence the consumers’ Engagement (H1) and Product perception (H2).

Answers to these research questions can offer actionable implications for future practice, illustrating how AR can transform packaging design into an interactive consumer touchpoint. The findings may also provide insights into the most effective consumer engagement strategies and help companies understand how to select and implement the most suitable type of functional AR for marketing among the various available typologies. Although this is an exploratory study, it is conceptually grounded in the assumption that the type of AR application, and specifically its degree of immersiveness, can influence both users’ engagement and product perception. These expected effects are represented in the following conceptual model (Figure 1), which illustrates the hypothesized relationships.

## 2. Methods

### 2.1. Sample

Twenty participants aged 25 to 60 years (M = 43.85, SD = 9.08) were recruited for the experimental study. The full sample was gender-balanced (10 males and 10 females) to reduce potential demographic biases. In addition, an attempt was made to balance each condition by gender and age, resulting in 5 people for each gender in each condition and a mean age of 45.80 (ds = 9.73) for the static condition and 41.90 (ds = 8.44) for the immersive condition; see Table 1). Since the dairy product label was used as the target stimulus, the inclusion criteria required that participants purchase dairy products and not be allergic to them. To assess study power, a post hoc sensitivity analysis was performed using G*Power 3.1.9.7 (Faul et al., 2009) based on a repeated-measures model (total sample size of 20, 1 group, 2 measurements, α = 0.05, 1 − β = 0.80, ρ = 0.5, ϵ = 1). It showed a minimum detectable effect size of f = 0.33, interpreted as “medium” to “large” (Cohen, 2013). This can be deemed adequate, considering the median effect size in cognitive neuroscience and experimental psychology of *d* = 0.93 (Szucs & Ioannidis, 2017), interpreted as more than “large” (Cohen, 2013). The sampling strategy adopted in this study is consistent with the recommendations outlined in the report by Clayson et al. (2025), which indicates that the typical number of participants per group in EEG studies is 21 (median = 18), as well as with previous EEG studies involving AR (Wimmer et al., 2025; Bosshard & Walla, 2023; Krugliak & Clarke, 2022; Garczarek-Bąk et al., 2021). The study was accepted by the university’s ethics committee and conducted in accordance with the Declaration of Helsinki (World Medical Association, 2013) and the General Data Protection Regulation. Before taking part in the experiment, participants signed an informed consent form to accept their participation in the study.

### 2.2. Materials

**Figure 2:**
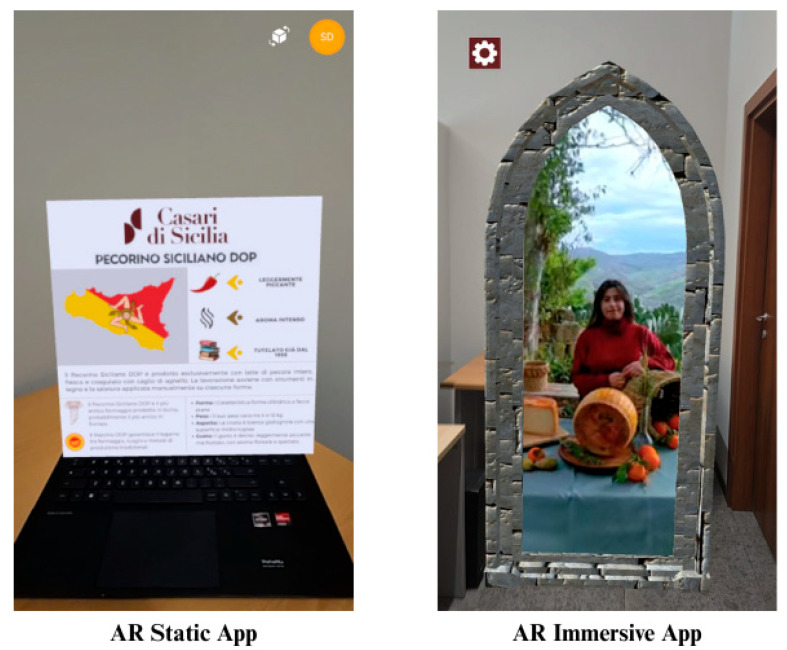
Augmented Reality applications used in the study. The left panel shows the AR application in its static version, while the right panel shows the same application in its immersive version.

Two AR apps with different types of interaction were developed for the study: static and immersive (Figure 2). The marker for the operation of the two apps was mounted on a cheese label, and a Samsung S24 was used for participants to interact with the apps. Both apps provide the same comprehensive details about the same cheese product, including its characteristics, history, and tradition. The only difference was that the information was delivered in different formats, inherently due to the nature of the two AR applications:

- Static AR: Content consisted of static pop-up image text above the label with details on the product. All information about the cheese was presented in written form and delivered passively, without any interaction with the augmented elements.
- Immersive AR: Content consisted of a virtual portal that appeared within the room. Users could walk through this portal to access a 360-degree video set inside a dairy farm. In the video, a dairy producer explained the product’s details. Participants had the opportunity to interact with the app by tapping predefined questions within the content, which triggered the corresponding video segment where the cheesemaker responded to the selected inquiry.

### 2.3. Experimental Design

The study employed a quantitative within-subjects experimental design in which all participants experienced both AR applications. A multi-modal measurement strategy combining implicit neurophysiological indices and explicit self-report scales was used to capture real-time and conscious evaluations of AR interaction.

Participants were randomly assigned and counterbalanced to start the study with either the static or the immersive AR condition, thereby ensuring that half of the sample commenced with the static AR and the remaining half with the immersive AR, to control for potential order effects. Randomization and counterbalancing were also applied across gender groups, ensuring that male and female participants were evenly distributed in the order of exposure to the static and immersive AR conditions. This procedure preserved the gender balance of the sample within each condition and minimized potential demographic biases. This counterbalancing ensured that any differences observed could not be attributed to the order of exposure. Randomization was also implemented to minimize possible learning, fatigue, or carryover effects that might otherwise influence the results. During the interaction, participants were free to explore each AR application without strict time constraints; however, if exploration exceeded five minutes, the researcher would gently invite them to proceed to the next task. Interactions with the AR content were carried out exclusively via smartphone. These procedural controls were specifically implemented to ensure comparability between conditions and to ensure that any observed differences could be attributed solely to the type of AR content.

### 2.4. Instrumentation

Since participants needed to move freely during the AR exploration, portable psychophysiological recording devices were used.

EEG data were collected through the X.on (Brain Products, GmbH, Gilching, Germany) headset from 7 Ag/AgCl semi-dry (0.09% NaCl water solution) electrodes placed on the scalp at standard 10-10 locations (F3, F4, C3, Cz, C4, P3, P4). The montage was monopolar, with reference and ground placed at the left earlobe (A1). The sample frequency was 500 Hz, and the vertical resolution was 24 bit. The EEG data were wirelessly streamed (BLE5 connectivity) to a recording PC running the Lab Streaming Layer (LSL) connector and recorded using the LSL’s LabRecorder App v1.16.4.

SC and PPG (photoplethysmography) data were collected through the Shimmer GSR+ (Shimmer Sensing, Ltd., Dublin, Ireland) at a sample frequency of 128 Hz and a vertical resolution of 16 bit. The SC was collected using 2 Ag/AgCl electrodes placed on the phalanxes of the index and middle fingers, while the PPG was collected from a reflective-type light sensor placed on the right earlobe (A2). The SC and PPG data were recorded on the Shimmer’s on-board SD card.

The eye-tracking (ET) data were collected through the Tobii Glasses 3 (Tobii AB, Stockholm, Sweden) device at a sample frequency of 50 Hz and with a 0.6° of angular accuracy. The ET data were recorded on the Recording Unit’s on-board SD card. The ET was used to synchronize neurophysiological data and extract the time intervals of the three tasks described below and the baselines. We used a TTL trigger with Tobii ET glasses using the Tobii controller software 1.19.4. After the researchers manually checked synchronization, the baseline phase began by inviting participants to fixate a white dot on a black background for 60 s with their eyes open and then close their eyes for 120 s. Furthermore, due to the inherent differences in content presentation between the static and immersive AR applications, quantitative ET data were excluded from the analysis. In the static condition, participants viewed a fixed image displaying textual information about the cheese, whereas in the immersive condition, the information was conveyed verbally by the cheesemaker within a 360-degree video. These substantial discrepancies in modality and visual layout rendered direct comparison of gaze metrics across conditions methodologically inappropriate. Event markers, necessary for analyzing EEG data during the tasks, were placed at the moment participants began interacting directly with the AR app and ended when they indicated that they had completed their exploration.

EEG, SC/PPG and ET recordings were started from the same PC, ensuring a common starting timestamp of all the devices. Using the Shimmer, the biological signals SC and HR were recorded via Consensys V1.6.0, and a joint analysis of SC and HR allowed the extraction of the Emotional Index (EI) (Vecchiato et al., 2014). Specifically, the study employed the X.on EEG with 7 channels. Signals were recorded via LabRecorder and processed with Matlab R2024b (EEGLab 2024.0.0). EEG metrics extracted included the Beta/Alpha + Theta Ratio (BATR) (Freeman et al., 1999; Fici et al., 2024) to measure the cognitive engagement related to visual attention. All the neurophysiological data were processed in Matlab following a standardized pipeline (Laureanti et al., 2021; Russo et al., 2023).

### 2.5. Neurophysiological Measures

#### 2.5.1. Emotional Index (EI)

The Emotional Index (EI) combines two physiological signals, skin conductance, reflecting emotional arousal (Dawson et al., 2007), and heart rate, associated with emotional valence (Nardelli et al., 2015), to synthesize an individual’s emotional experience. The EI is constructed on a bipolar scale, with positive values (greater than zero) indicating a positive emotional response and negative values reflecting a predominantly negative response. The EI provides an effective measure of emotional engagement during consumer experiences (Vecchiato et al., 2014), enabling the assessment of information acquisition processes through affective physiological responses in both traditional packaging (Modica et al., 2018) and digital experiences (Fici et al., 2024). The EI enables a comprehensive assessment of the user’s psychophysiological activation, both during active interaction and passive observation. In the context of AR experiences, where elements of immersiveness or static graphical overlays may be involved, evaluating how these different AR typologies influence users’ psychophysiological responses is critical. Examining the level of emotional engagement elicited by the two types of AR applications provides critical insight that allows for a deeper understanding of consumer behavior during the interaction with different forms of AR, ultimately shedding light on how specific AR formats may shape affective processing in marketing contexts.

#### 2.5.2. Beta/Alpha Theta Ratio (BATR)

The Beta over Alpha plus Theta Ratio (BATR) is an EEG-based indicator developed by Pope et al. (1995) to monitor cognitive engagement. It is calculated by averaging the beta power to the total of the alpha and theta powers for each electrode. The BATR is based on a continuous scale, where positive values (exceeding zero) signify favorable engagement, and negative values denote primarily unfavorable responses. BATR provides a complementary measure to emotional indices, specifically assessing cognitive involvement, particularly in tasks requiring visual attention when interacting with digital environments (Fici et al., 2024). For this reason, the use of the BATR index represents the most appropriate choice for analyzing AR interactions, as it enables the direct assessment of cognitive engagement during human–machine interaction. In particular, the interaction mediated by the smartphone requires users to allocate attentional resources, a process that the BATR is specifically suited to detect and quantify (Pope et al., 1995; Freeman et al., 1999). Analyzing user interaction with different types of AR from a cognitive perspective provides valuable insights into consumer psychology by revealing how certain AR formats may elicit higher levels of cognitive involvement than others.

### 2.6. Self-Report Measures

Participants were administered two self-report measurement scales via an online platform, as presented in Table 2.

The Augmented Reality Immersion (ARI) scale (Georgiou & Kyza, 2017) was used to measure different aspects of immersiveness in the two AR applications by analyzing the macrodimensions of Engagement (8 items), Engrossment (6 items), and Total Immersion (7 items). This instrument was specifically used to assess the level of immersiveness of the apps.

The Perceived Informativeness (PI) scale (Holdack et al., 2022) was used to measure how informative AR applications with static or immersive content are perceived by users (3 items).

Perceived Brand Authenticity (PBA) (Park et al., 2021) was used in its original form to assess the extent to which AR applications enhanced consumers’ perception of brand authenticity and in an adapted form to evaluate the Perceived Product Authenticity (PPA) (3 items).

Finally, Intention to Buy (ITB) (Russo et al., 2021) was employed to assess the extent to which the type of AR application could influence consumers’ willingness to purchase the product promoted through AR (3 items).

All items were measured on a 7-point Likert scale from 1 (strongly disagree) to 7 (strongly agree). The scales were translated into the target language following the back-translation procedure (Brislin, 1970) to ensure linguistic and conceptual equivalence.

### 2.7. Protocol

The assembly phase began after participants were welcomed by the laboratory staff and signed the informed consent. The EEG, HR, and SC sensors were placed on the participant. Before the experimental tasks, participants first took a 60 s eyes-closed (EYC) period to record a resting-state baseline. This was followed by a second baseline (BSL), during which participants were instructed to keep their eyes open and fixate on a white dot displayed on a black background for 120 s. These baseline recordings were used for data processing.

At the beginning of the experimental tasks, the participants randomly interacted with cheese labels in which AR applications were implemented. The labels were visualized in front of a 21.5″ PC monitor (P2217H by DELL). The experimentation was divided into two phases:

- Task 1: The participant pointed the packaging label through the cell phone camera and interacted with the AR application using a smartphone for up to 5 min, for as long as they thought it was appropriate.
- Task 2: After the AR interaction, one of the two researchers accompanied the participant in completing a questionnaire on the AR interaction. The questionnaire lasted an average of 7 min.

**Figure 3:**
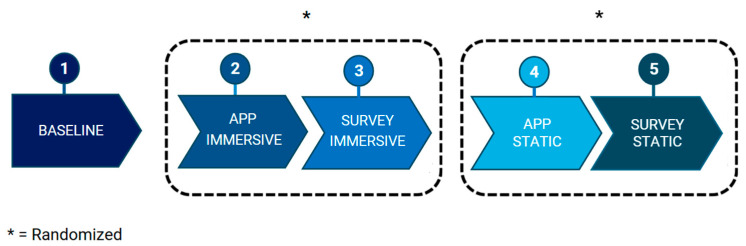
Experimental protocol sequence of the study. The figure illustrates the sequence of phases: baseline recordings, interaction with the AR applications (static and immersive, with counterbalanced order across participants), and completion of the self-report questionnaires.

After completing the questionnaire, the participant returned to the initial position to start the task again. This procedure was repeated two times until participants had interacted with both versions of AR (Figure 3).

### 2.8. Data Processing

EEG, SC, and PPG data were processed in the MATLAB environment (The MathWorks, Inc., Natick, MA, USA). First, the data were aligned to a common timeline using the individual timestamps. Then, the video recordings were examined, and markers corresponding to the onset and offset of each task (i.e., experimental phases and baselines) were placed. These were subsequently exported and appended to the data.

The EEG was processed using the EEGLab (Delorme & Makeig, 2004) toolbox. Slow voltage drifts and high frequency noise were attenuated by a band-pass filter (0.1–40 Hz zero-phased IV order Butterworth filter), and the power line interference was filtered by means of the CleanLine (Bokil et al., 2010) multi-taper regression (50 and 100 Hz). Non-stationary artefacts were corrected by means of the Artefact Subspace Reconstruction method (Chang et al., 2018) with standard cut-off values (k = 10). Then, stereotypical artefacts were corrected by means of Independent Component Analysis (FastICA algorithm—Hyvärinen & Oja, 2000). Specifically, Artefactual Independent Components (ICs) were automatically identified using ICLabel (Pion-Tonachini et al., 2019) as those with “not-brain” probability, *p* > 0.9, and removed. Non-artefactual ICs were, thus, back-projected to the original sensor space. Finally, the cleaned EEG was re-referenced to the theoretically desired zero-potential using the REST algorithm (Dong et al., 2017), which has been shown to be particularly effective even for reduced electrode coverage (Hu et al., 2018).

For each subject, the Individual Alpha Frequency (IAF) was estimated as the center of gravity (Klimesch, 1997) of the Power Spectral Densities (PSDs) averaged across P3 and P4 channels. The PSDs were computed following the Welch’s method (1 s-long Hamming window and 50% of overlapping—Bilucaglia et al., 2019), considering the EYC baseline. The IAF served to define the following subject-specific EEG bands: *ϑ* = [IAF − 6, IAF − 2], *α* = [IAF − 2, IAF + 2], and *β* = [IAF + 2, IAF + 26] (Borghini et al., 2019).

The BATR was computed as the ratio between *β* and +*α* instant powers averaged over the entire channel set. The power computation followed the spectrographic approach (1 s long Hamming window and 50% of overlapping) with normalization (Bilucaglia et al., 2021). BATR consisted of a temporal signal with 0.5 s of temporal resolution.

The SC was downsampled to 32 Hz. High-frequency noise was attenuated by a low-pass filter (0.35 Hz zero-phased IV order Butterworth filter). Then, artefactual points were identified through a double threshold method (0.05–60 μS, ±10 μS/s), deleted and linearly interpolated from neighbor data (2 s-long centered window—Kleckner et al., 2017). The tonic SC level (SCL) was finally obtained using the cvxEDA algorithm (Greco et al., 2015).

The PPG was downsampled to 32 Hz. High frequency noise was attenuated by a low-pass filter (5 Hz zero-phased IV order Butterworth filter), while the baseline drift was corrected through a deconvolution method based on the Hilbert-estimated envelope (Dall’Olio et al., 2020). PPG peaks corresponding to the maximum blood perfusion were identified by means of the AMPD algorithm (Scholkmann et al., 2012). The HR was finally computed by smoothing (2 s-long moving average filter) the inverted peak-to-peak temporal distances.

The EI index was computed by applying the 2-arguments arctangent function on SLC and HR signals, resulting in a temporal signal with 1/32 s of resolution. To obtain a condensed stimulus-related index, BATR and EI signals were epoched according to the experimental tasks, temporally averaged, and z-scored according to the mean and standard deviation within the BSL epoch (Russo et al., 2022a).

All neurophysiological indices (BATR and EI) were baseline-corrected using the reference periods collected before the experimental tasks, ensuring that subsequent analyses reflected changes relative to each participant’s resting state.

### 2.9. Statistical Analysis

Statistical analysis was performed using JASP v. 0.19, an R-based statistical software package (Love et al., 2019). All the items analyzed were averaged, and the internal reliability was assessed using both Cronbach’s alpha (Nunnally, 1978) and McDonald’s Omega (McDonald, 1999) coefficients for each construct (Table 3). The Shapiro–Wilk test for normality was conducted to assess the assumptions of normal distribution for both neurophysiological and self-report measures. Most variables did not significantly deviate from a normal distribution. For the few variables that showed non-normality (e.g., I_PBA, I_PPA, I_EI; Table 4), parametric tests were still applied, given their robustness in moderately sized samples (N = 20), and results were verified with non-parametric equivalents where relevant. To assess the study hypotheses, a two-way repeated measures ANOVA examined differences in neurophysiological indicators (two levels: BATR, EI) and AR content (two levels: static and immersive). For ARI constructs, an RM ANOVA (three levels: Engagement, Engrossment, and Total Immersion) was employed, considering AR content (two levels: static and immersive) as a factor. Finally, for other self-report measures, an RM ANOVA (three levels: PI, PBA, PPA and ITB) was employed, considering AR content (two levels: static and immersive) as a factor.

For both ANOVAs, Mauchly’s test checked sphericity, with a Greenhouse–Geisser correction applied if it was violated. All post hoc comparisons reported were adjusted using Holm’s correction for multiple testing. To verify the robustness of the results, the Friedman test was conducted for variables that did not meet the assumption of normality, comparing the static and immersive conditions. Finally, Pearson’s correlations were conducted for each AR content type, correlating neurophysiological indicators (BATR and EI) with ARI and other self-report data. Although not designed to test the study hypotheses directly, they provide the additional interpretative value of both converging and diverging patterns across explicit and implicit measures. To improve transparency, the full correlation matrix per condition was reported. The significance level was set at α = 0.05.

Considering the relatively limited sample size, the findings should be regarded as exploratory and not extrapolated to the broader population. Nonetheless, they offer valuable preliminary insights and establish a foundation for subsequent research in this field.

## 3. Results

### 3.1. Neurophysiological Results

**Figure 4:**
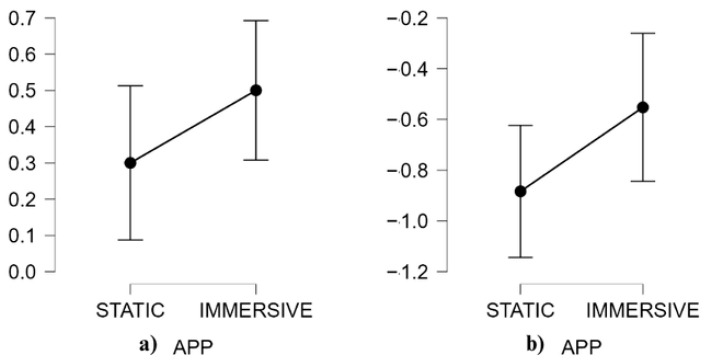
Neurophysiological descriptive plots with 95% CI error bars of EI (**a**) and BATR (**b**) split according to AR content (static and immersive).

Table 5 and Figure 4 present descriptive data (means, M, and standard deviations, SD) and descriptive plots with 95% confidence interval bars for neurophysiological results.

The results from neurophysiological signals showed a significant main effect of AR Content, *F*(1, 19) = 8.54; *p* = 0.009; η^2^ = 0.03; ω^2^ = 0.06, and of neurophysiological, *F*(1, 19) = 54.58; *p* < 0.001; η^2^ = 0.60; ω^2^ = 0.50. There is no significance of neurophysiological × AR content interaction. Although the interaction was not significant, exploratory post hoc comparisons were conducted to better understand specific differences between static and immersive AR conditions, given the study’s exploratory nature. The S content and I content showed a significant difference in favor of the immersive app for EI, (*t*(19) = −2.76, MD = −0.20, SE = 0.07, *p* = 0.025, *d* = −0.32). The result was confirmed by a non-parametric analysis conducted on the EI, which did not meet the assumption of normality. Also, the non-parametric Friedman test revealed a significant difference between conditions (T = 2.14, df = 57, *p* = 0.037, *r_s_* = −0.61). The I content condition showed a marginally significant increase in BATR compared to the S condition (*t*(19) = −1.93, MD = −0.33, SE = 0.17, *p* = 0.069, *d* = −0.52).

### 3.2. ARI Results

**Figure 5:**
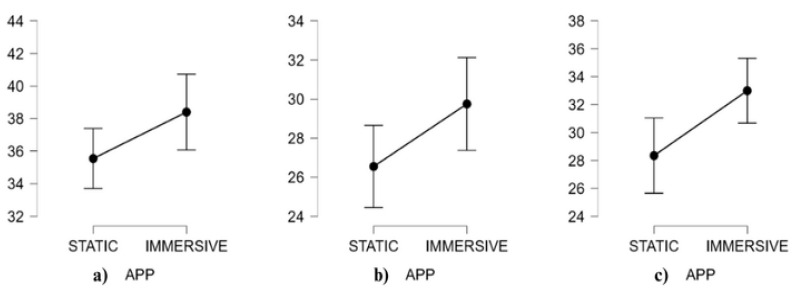
Augmented Reality Immersion constructs plots with 95% CI error bars of Engagement (**a**), Engrossment (**b**), and Total Immersion (**c**) split according to AR content (static and immersive).

Table 6 and Figure 5 present descriptive data (means, M, and standard deviations, SD) and descriptive plots with 95% confidence interval bars of ARI questionnaire results.

The results from the ARI scale showed a significant main effect of AR content, *F*(1, 19) = 11.29, *p* = 0.003, η^2^ = 0.09, ω^2^ = 0.05, and of ARI constructs, *F*(1, 19) = 27.67, *p* < 0.001, η^2^ = 0.38, ω^2^ = 0.17. There is no significance in the AR content × ARI constructs interaction. Although the interaction was not significant, exploratory post hoc comparisons were conducted to better understand specific differences between static and immersive AR conditions, given the study’s exploratory nature. The S content and I content showed a significant difference in favor of the immersive app for the Total Immersion construct, *t*(19) = −3.48, MD = −4.65, SE = 1.34, *p* = 0.020, *d* = −0.54. The result was confirmed by a non-parametric analysis conducted on the Total Immersion, which did not meet the assumption of normality. Also, the non-parametric Friedman test revealed a significant difference between conditions (T = 3.27, df = 95, *p* = 0.001, *r_s_* = −0.78).

### 3.3. Self-Report Results

**Figure 6:**
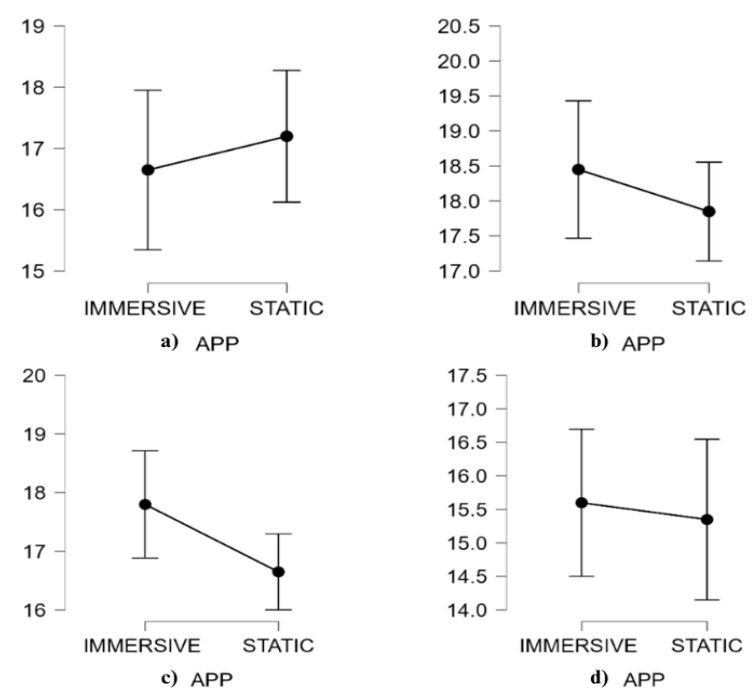
Self-report plots with 95% CI error bars of PI (**a**), PBA (**b**), PPA (**c**), and ITB (**d**) split according to AR content (static and immersive).

Table 7 and Figure 6 present descriptive data (means, M, and standard deviations, SD) and descriptive plots with 95% CI bars of other self-report results.

The results from the self-report questionnaire did not show a significant main effect on AR content, but the effect was significant on self-report, *F*(1, 19) = 147.67, *p* = 0.004, η^2^ = 0.19, ω^2^ = 0.07, and AR content × self-report, *F*(1, 19) = 15.22, *p* = 0.032, η^2^ = 0.02, ω^2^ = 0.01.

Analyzing the post hoc comparisons revealed no significance among the interactions. Even the non-parametric test did not reveal any significant statistical results.

### 3.4. Correlation Results

To assess correlations between the variables, Pearson’s correlation was employed, due to the fact that the majority of the variables met the normality assumption. Pearson’s correlation analysis revealed positively significant associations between ARI constructs and self-report measures but not with neurophysiological variables. In the static AR condition, PI positively correlated with Engagement (*ρ* = 0.64, *p* = 0.002) and Engrossment (*ρ* = 0.46, *p* = 0.040). Additionally, PI was positively correlated with both PBA (*ρ* = 0.72, *p* < 0.001) and PPA (*ρ* = 0.63, *p* = 0.003). Finally, ITB was positively correlated with all the dimensions analyzed (see Table 8). In the immersive AR condition, PI was positively correlated with Total Immersion (*ρ* = 0.48, *p* = 0.034). Engagement was positively correlated with both PPA (*ρ* = 0.55, *p* = 0.012) and PBA (*ρ* = 0.54, *p* = 0.015). Finally, ITB was positively associated with all measured dimensions, except for Total Immersion and PI (see Table 9). The measures related with the same scale are statistically correlated, as shown in Table 7 and Table 8.

## 4. Discussion

This exploratory study investigated the impact of two different mobile AR modalities—static and immersive—applied to food packaging labels, evaluating their effects on consumer experience. To do so, a within-subjects experimental design was adopted on a sample of 20 participants, integrating neuroscientific measures (EEG, HR, SC) with self-report questionnaires. The aim was to investigate how the two types of mobile AR content influence several variables, such as cognitive and emotional engagement, immersion, informativeness, product and brand authenticity, and purchase intention. The study additionally examined the correlations between these variables in both conditions to offer further interpretive insights for the research. Table 10 summarizes the answers to all the research hypotheses addressed in this study.

### 4.1. Immersive AR Engages Emotionally and Cognitively

The neurophysiological results suggest that immersive AR generates higher consumer engagement levels from a neurophysiological perspective compared to the static AR. Specifically, participants experienced a statistically significant greater emotional engagement during the immersive AR experience than with the static AR content, as measured by the EI through HR and SC. From a cognitive perspective, the results partially corroborate our hypothesis. Although both experiences negatively impacted cognitive engagement, as measured by the BATR through EEG, the immersive AR condition was rated as less negative than the static one. However, this difference did not reach statistical significance, though the trend points in that direction. Therefore, in relation to the first research question (RQ1), the neurophysiological data indicate a significant difference between the static and immersive apps in terms of emotional response, and a close to significant difference in cognitive involvement. The application’s immersive role was also confirmed from a rational point of view, as evidenced by the statistically significant difference found in the Total Immersion construct of the ARI questionnaire between the two conditions, although the other ARI constructs, particularly Engrossment and Engagement, did not show the same difference. This result supports the conceptual distinction between the two applications in terms of content typology and interaction, distinguishing between immersive and non-immersive formats. On the other hand, self-report results show no difference between immersive and static apps in the measured constructs of PI, PBA, PPA, and ITB. This suggests that while the immersive AR may enhance the perceived depth of the experience, this is not necessarily reflected in the reported consumer attitudes towards the product or the brand. Therefore, the questionnaire results do not definitively confirm the second research question (RQ2). This outcome is consistent with the findings of Pozharliev et al. (2022), who, in comparing self-reported and physiological arousal responses to AR advertisements versus traditional ads, found that only physiological measurements could detect a higher level of appreciation and a greater willingness to pay for AR ads. It highlights how neurophysiological and self-reported data do not always align, reinforcing the importance of a multimodal approach. This divergence between heightened emotional arousal and the absence of corresponding differences in attitude or behavioral intent may be interpreted through the lens of dual-process theories of persuasion. According to the ELM (Petty & Cacioppo, 1986), persuasive messages can follow a central route, requiring cognitive elaboration, or a peripheral route, driven more by affective responses. In our case, immersive AR appears to stimulate stronger affective reactions (peripheral route), yet these do not necessarily translate into cognitive and rational evaluations or intentions (central route). This theoretical framework helps explain why physiological engagement can emerge without changes in reported outcomes, highlighting the challenge of bridging emotion, perception, and action in consumer decision-making.

More generally, the neurophysiological findings are consistent with earlier research highlighting the importance of immersive AR content attributes in enhancing users’ overall engagement (Scholz & Smith, 2016). Indeed, regarding emotional engagement, the results align with previous research showing that the degree of immersion and interactivity enhances consumers’ enjoyment (Yim et al., 2017; Raman et al., 2025) and emotional engagement (Nhan et al., 2022), suggesting that immersive AR applications foster stronger affective resonance in consumers during the first interaction with the product. Features such as interactivity and 360-degree video, an immersive form of dynamic AR integrated into the mobile AR experience, allow consumers to virtually enter the place of production of dairy cheese and hear the producer’s own words about the product, contributing to a vivid, emotionally engaging user experience. This approach enabled consumers not only to gather information about the product but also to connect with it on an emotional level. From a theoretical standpoint, these findings support and extend Flow Theory (Nakamura & Csikszentmihalyi, 2009), suggesting that immersive environments can foster deeper emotional absorption, even in consumer scenarios like product packaging. Additionally, the results are consistent with Media Richness Theory (Tseng & Wei, 2020), indicating that the richness of AR media—through its multimodal and interactive elements—enhances users’ experience and emotional engagement, which in turn can influence decision-making (de Amorim et al., 2022). Collectively, these insights underscore the potential of immersive AR not merely as a functional communication tool, but as an affective medium—an important consideration for future research and design in Human–Computer Interaction. Regarding cognitive engagement, the results are partially in line with prior research, which shows that the degree of immersion is positively associated with increased neural activity in brain regions involved in cognitive functions, such as attention (Xu & Sui, 2021) and working memory (Souza & Naves, 2021). It could be explained by the fact that the combination of multimodal elements, in particular storytelling (Sung et al., 2023), can enhance cognitive processing and engagement (Mayer, 2005; Farida & Clark, 2024), and multisensory stimuli can lead to stronger cognitive and neural processing (Stein & Stanford, 2008). However, considering the low cognitive engagement in both conditions and the lack of statistically significant differences between them, it is worth noting that, as Javornik (2016) points out, emotional engagement plays a more critical role than cognitive engagement in shaping consumer responses, particularly during initial interactions with AR. It suggests that the emotional dimension, even in the absence of extensive cognitive processing, can be a valuable mechanism in influencing consumer responses, particularly in early-stage interactions, where affective reactions often guide attitude and decision-making. In this regard, the ELM (Petty & Cacioppo, 1986) provides a valuable interpretative framework for persuasive messages, suggesting that users’ interaction with the immersive application predominantly engages affective responses through the peripheral route, rather than fostering deliberate cognitive elaboration via the central route. Moreover, the correlation analysis provides valuable insights into the relationship between different forms of AR content and consumer perceptions and behavioral intentions. Indeed, these were not meant as confirmatory tests but as exploratory analyses designed to offer a more detailed view of the relationships across methodologies in relation to the two types of AR. First, a key finding is that self-report measures were consistently interrelated, whereas neurophysiological indices did not show significant correlation with them. This still confirms a partial dissociation between implicit (physiological) and explicit (self-reported) responses, as already noted in prior research (Pozharliev et al., 2022), and underscores the importance of adopting multimodal methodologies in consumer neuroscience. Interestingly, the two types of AR content, when considered separately, demonstrated distinct correlation patterns with self-reported consumer perception. In the static AR condition, PI was positively associated with both PBA and PPA, as well as with Engagement and Engrossment. This suggests that when AR content is static and primarily fixed information-driven, consumers’ perception of informativeness can act as a central mediator, reinforcing their evaluations of the product and the experience. In contrast, in the immersive AR condition, Engagement was the key variable positively associated with PBA and PPA. This highlights that in immersive contexts, consumers’ sense of being actively engaged with the content could drive perceptions of authenticity. Importantly, both AR applications demonstrated positive correlations between ITB and most of the measured dimensions, indicating that purchase intention in AR contexts is supported by a broad network of factors, ranging from informativeness and authenticity to the subjective feeling of engagement. Finally, this study offers preliminary evidence to the field of consumer neuroscience in the context of AR by providing both neurophysiological and self-reported data that immersive AR, when applied to food packaging, could be more effective to improve the quality of the user experience than static AR.

### 4.2. Managerial Implications

Such analysis is relevant not only from a scientific perspective for understanding the underlying mechanisms of AR technology, but also from a managerial standpoint, as it provides potentially useful insights for designing more effective AR-based marketing strategies. Indeed, from a managerial perspective, the findings provide preliminary information that can inform companies how to approach the use of AR on packaging. Although the evidence is exploratory and based on a small sample, the results suggest that immersive AR may foster stronger emotional engagement than static AR, while correlation evidence shows how different AR formats could influence consumer perceptions. Managers should therefore view AR applications on packaging not as universally effective tools, but as context-dependent strategies whose impact may vary according to the type of content. Rather than assuming immersive AR will always lead to more favorable outcomes, brands could consider pilot-testing different AR formats to identify which experiences best align with their communication objectives and target audiences. Thus, the specific type of AR content should be strategically selected based on the consumer experience they aim to foster. The adoption of immersive AR applications should not be confined to the B2C (Business to Consumer) domain but may also generate substantial value in B2B (Business to Business) contexts. Specifically, their implementation during national and international food industry events could provide companies with an emotionally engaging experience by virtually immersing them in the territory where the raw material is cultivated and transformed into the final product. Through such mediated immersion, firms are enabled not only to perceive but also to experience the production environment, thereby strengthening authenticity perceptions and fostering deeper connections with the brand narrative. In this sense, the study highlights the importance of carefully tailoring AR investments and of treating current findings as indicative directions that warrant further validation in real-world retail environments.

### 4.3. Limitations and Future Directions

This exploratory study allowed the assessment of the actual differences in consumer interaction with AR when implemented on packaging labels. However, it is not without limitations. The sample size is thought to be suitable for preliminary research and neurophysiological analysis, but it might have an impact on how broadly the findings can be applied. The near to significant result observed in the BATR may be attributed to the limited sample size; with a larger sample, statistical significance might be achieved. The authors recommend conducting further research with a larger participant sample, which would consequently increase the statistical power of the study. Participants’ familiarity or prior experience with Augmented Reality was neither considered as an inclusion criterion nor systematically assessed during the experiment, which may have influenced the novelty effect and, consequently, the level of cognitive engagement. Although the global number of AR users is rapidly increasing, estimated at 1.07 billion (Statista, 2024), it will be important for future research to incorporate a measure of AR familiarity. Ideally, this would involve the use, or development, of a dedicated scale specifically tailored to AR applications in marketing contexts, to more accurately account for individual differences in prior experience and technological competence. Moreover, artificial immersion in a laboratory setting clearly allows for observing user responses without external influences, but it also overlooks the social context of a supermarket and the actual shopping habits of consumers. Therefore, conducting future studies in real environments such as retail stores or supermarkets would not only enhance ecological validity but also increase the generalizability of the findings. Additionally, participants used the Samsung S24 smartphone, which, despite having one of the largest screens among smartphones, may limit the enjoyment of the experience. Consequently, using the latest generation of Augmented Reality tools or glasses could provide further insights from the research. A further limitation is that the use of actual packaging was not possible during the study. Introducing an additional condition in which participants interact directly with traditional packaging—without the mediation of a digital device—would enhance our understanding of human interaction with AR in consumer contexts. Such a control condition would allow for the assessment of product interaction in the absence of any digital augmentation. Finally, the AR applications used in this study were designed without gamification elements or high levels of user interaction. Users were required only to read information in the static condition and to watch a 360-degree video, selecting the questions they wished to ask, in the immersive condition. Future research could investigate the impact of these features from an authenticity perspective and through neurophysiological measures, offering companies valuable insights into the most effective strategies for their marketing campaigns.

## 5. Conclusions

In conclusion, this exploratory study provides preliminary indications of experiential and neurophysiological difference between static and immersive apps when embedded within product packaging labels. Results suggest that immersive AR may elicit higher emotional engagement, while evidence on cognitive engagement remains less conclusive. Thus, unlike static AR that mainly delivers fixed product-related information, immersive AR provides richer contextual cues that could foster a more engaging consumer experience. Moreover, the correlation results revealed distinct patterns for the two AR typologies: static AR primarily enhanced authenticity through Perceived Informativeness, whereas immersive AR did so through engagement. Although the small sample size limits the generalizability of the findings, the results provide preliminary evidence that different types of AR may shape consumer experience in distinct ways during interaction. For companies, these distinctions may be relevant, as they offer directions that could support the development of marketing strategies tailored to specific campaign goals. The insights, while exploratory, can still inform companies in considering how various AR formats might be aligned with specific communication and campaign objectives.

This study contributes to opening new research directions in the literature on consumer neuroscience and consumer interaction with AR, particularly in the context of retail packaging. Understanding consumer engagement and physiological responses becomes increasingly crucial as AR technology becomes more integrated into packaging strategies. Given the exploratory nature of this research, future steps will involve a deeper analysis of consumers’ cognitive and affective reactions to packaging interaction. Expanding the investigation to additional product categories will allow the generalization of findings beyond dairy products. Furthermore, testing a condition without digital interaction, focused solely on traditional packaging in real-world retail environments such as supermarkets, would enhance ecological validity. Considering the inherently interactive nature of AR, future research should also explore the integration of gamification elements, which could not only shed light on their impact on users’ cognitive and emotional responses but also provide companies with clear guidelines for investing in augmented labels. In doing so, AR applications may offer consumers additional reasons for engagement, ultimately influencing perceptions of both brand and product authenticity.

## Figures and Tables

**Table 1:** Demographic characteristics (gender and age) for each condition.

Demographic Characteristics			
Variables	Category	Static AR	Immersive AR
Gender	Male	5	5
	Female	5	5
Age	Mean	45.80	9.73
	Dev. stand.	41.90	8.44

**Table 2:** Constructs and corresponding items used in the experiment.

Constructs	Item *	References
Engagement	I liked the activity because it was novel	Adopted and revised from Augmented Reality Immersion questionnaire (Georgiou & Kyza, 2017)
	I liked the type of the activity	
	I wanted to spend the time to complete the activity successfully	
	I wanted to spend time to participate in the activity	
	It was easy for me to use the AR application	
	I found the AR application confusing	
	The AR application was unnecessarily complex	
	I did not have difficulties in controlling the AR application	
Engrossment	I was curious about how the activity would progress	
	I was often excited since I felt I was part of the activity	
	I often felt suspense in the activity	
	If interrupted, I looked forward to returning to the activity	
	Everyday thoughts and concerns faded out during the activity	
	I was more focused on the activity rather on any external distraction	
Total Immersion	The activity felt so authentic that it made me think that the virtual objects existed for real	
	I felt that what I was experiencing was something real, instead of a fictional activity	
	I was so involved in the activity that in some cases I wanted to interact with the virtual objects directly	
	I so was involved that I felt that my actions could affect the activity	
	I did not have any irrelevant thoughts or external distractions during the activity	
	The activity became the unique and only thought occupying my mind	
	I lost track of time, as if everything just stopped, and the only thing that I could think about was the activity	
Perceived Informativeness	The AR app provides complete information about the cheese	Adopted and revised from (Holdack et al., 2022)
	The AR app provides information that helps me in my buying decision	
	The AR app provides information to compare products.	
Perceived Brand Authenticity	The brand of the app is genuine	Adopted and revised from (Park et al., 2021)
	The brand of the app is authentic	
	The brand of the app is real	
Perceived Product Authenticity	The product of the app is genuine	
	The product of the app is authentic	
	The product of the app is real	
Intention to Buy	I would like to try this product	Adopted and revised from (Russo et al., 2021)
	I would buy this product if I happened to see it	
	I would actively seek out this product in a store in order to purchase it	

* All items were measured on a 7-point Likert scale anchored from 1 (strongly disagree) to 7 (strongly agree).

**Table 3:** Internal reliability of the constructs (Cronbach’s α and McDonald’s ω).

Constructs	N. Item	M	SD	Cronbach’s α	McDonald’s ω
Engagement	8	36.98	7.08	0.68	0.76
Engrossment	6	28.15	9.01	0.93	0.94
Total Immersion	7	30.68	9.71	0.86	0.86
Perceived Informativeness	3	16.93	2.90	0.60	0.61
Perceived Brand Authenticity	3	18.15	2.99	0.91	0.93
Perceived Product Authenticity	3	17.23	3.25	0.84	0.85
Intention to Buy	3	15.48	4.07	0.90	0.91

**Table 4:** Results of the Shapiro–Wilk normality tests for all dependent variables, reported separately for static and immersive AR conditions.

Condition	Static	Immersive
Dependent Variables	*p*-Value of Shapiro-Wilk	*p*-Value of Shapiro-Wilk
Engagement	0.540	0.180
Engrossment	0.158	0.248
Total Immersion	0.326	0.849
Perceived Informativeness	0.167	0.088
Perceived Brand Authenticity	0.002	0.001
Perceived Product Authenticity	0.280	0.002
Intention to Buy	0.001	0.004
Emotional Index	0.270	0.006
BATR	0.431	0.306

**Table 5:** Neurophysiological descriptive statistics (mean—M, standard deviation—SD, CI—confidence interval) of EI and BATR split according to AR content (static and immersive).

	BATR				EI			
			95% CI				95% CI	
AR Content	M	SD	Min	Max	M	SD	Min	Max
Static	−0.88	0.75	−1.23	−0.53	0.30	0.37	0.13	0.47
Immersive	−0.55	0.88	−0.97	−0.14	0.50	0.36	0.33	0.67

**Table 6:** Augmented Reality Immersion constructs descriptive statistics (mean—M, standard deviation—SD, CI—confidence interval) of Engagement, Engrossment, and Total Immersion split according to AR content (static and immersive).

	Static				Immersive			
			95% CI				95% CI	
Constructs	M	SD	Min	Max	M	SD	Min	Max
Engagement	35.55	6.68	32.43	38.68	38.40	7.36	34.96	41.84
Engrossment	26.55	9.20	22.24	30.86	29.75	8.76	25.65	33.85
Total Immersion	28.35	9.46	23.93	32.78	33.00	9.64	28.49	37.5

**Table 7:** Self-report descriptive statistics (mean—M, standard deviation—SD, CI—confidence interval) of PI, PBA, PPA, and ITB split according to AR content (static and immersive).

	Static				Immersive			
			95% CI				95% CI	
Constructs	M	SD	Min	Max	M	SD	Min	Max
Perceived Informativeness	17.20	2.82	15.88	18.52	16.65	3.01	15.24	18.06
Perceived Brand Authenticity	17.85	2.83	16.52	19.18	18.45	3.19	16.96	19.94
Perceived Product Authenticity	16.65	3.13	15.18	18.12	17.80	3.35	16.23	19.37
Intention to Buy	15.35	4.30	13.34	17.36	15.60	3.94	13.76	17.44

**Table 8:** Correlation matrix of static condition between self-report and neurophysiological data.

Correlation Matrix—Static Condition										
		Engagement	Engrossment	Total Immersion	PI	PBA	PPA	ITB	EI	BATR
Engagement	Pearson’s r	—								
	df	—								
	*p*-value	—								
Engrossment	Pearson’s r	0.854 ***	—							
	df	18	—							
	*p*-value	<0.001	—							
Total Immersion	Pearson’s r	0.685 ***	0.716 ***	—						
	df	18	18	—						
	*p*-value	<0.001	<0.001	—						
PI	Pearson’s r	0.642 **	0.462 *	0.441	—					
	df	18	18	18	—					
	*p*-value	0.002	0.040	0.051	—					
PBA	Pearson’s r	0.430	0.389	0.153	0.722 ***	—				
	df	18	18	18	18	—				
	*p*-value	0.058	0.090	0.519	<0.001	—				
PPA	Pearson’s r	0.369	0.520 *	0.292	0.634 **	0.776 ***	—			
	df	18	18	18	18	18	—			
	*p*-value	0.109	0.019	0.211	0.003	<0.001	—			
ITB	Pearson’s r	0.547 *	0.735 ***	0.539 *	0.567 **	0.588 **	0.803 ***	—		
	df	18	18	18	18	18	18	—		
	*p*-value	0.013	<0.001	0.014	0.009	0.006	<0.001	—		
EI	Pearson’s r	0.015	0.002	0.030	0.293	0.140	0.180	−0.078	—	
	df	18	18	18	18	18	18	18	—	
	*p*-value	0.949	0.992	0.899	0.211	0.557	0.449	0.745	—	
BATR	Pearson’s r	0.212	0.356	−0.175	0.113	0.421	0.391	0.382	0.151	—
	df	18	18	18	18	18	18	18	18	—
	*p*-value	0.370	0.123	0.461	0.637	0.064	0.089	0.097	0.524	—

Note. * *p* < 0.05, ** *p* < 0.01, *** *p* < 0.001.

**Table 9:** Correlation matrix of immersive condition between self-report and neurophysiological data.

Correlation Matrix—Immersive Condition										
		Engagement	Engrossment	Total Immersion	PI	PBA	PPA	ITB	EI	BATR
Engagement	Pearson’s r	—								
	df	—								
	*p*-value	—								
Engrossment	Pearson’s r	0.732 ***	—							
	df	18	—							
	*p*-value	<0.001	—							
Total Immersion	Pearson’s r	0.588 **	0.782 ***	—						
	df	18	18	—						
	*p*-value	0.006	<0.001	—						
PI	Pearson’s r	0.351	0.312	0.475 *	—					
	df	18	18	18	—					
	*p*-value	0.129	0.181	0.034	—					
PBA	Pearson’s r	0.537 *	0.276	0.021	0.258	—				
	df	18	18	18	18	—				
	*p*-value	0.015	0.239	0.931	0.271	—				
PPA	Pearson’s r	0.552 *	0.280	0.080	0.269	0.916 ***	—			
	df	18	18	18	18	18	—			
	*p*-value	0.012	0.232	0.738	0.251	<0.001	—			
ITB	Pearson’s r	0.707 ***	0.595 **	0.359	0.271	0.648 **	0.748 ***	—		
	df	18	18	18	18	18	18	—		
	*p*-value	<0.001	0.006	0.120	0.247	0.002	<0.001	—		
EI	Pearson’s r	−0.227	−0.063	−0.020	−0.120	−0.097	0.010	−0.198	—	
	df	18	18	18	18	18	18	18	—	
	*p*-value	0.337	0.792	0.932	0.614	0.685	0.966	0.404	—	
BATR	Pearson’s r	0.316	0.274	0.115	0.023	0.178	0.180	0.076	0.369	—
	df	18	18	18	18	18	18	18	18	—
	*p*-value	0.174	0.242	0.630	0.924	0.452	0.448	0.749	0.109	—

Note. * *p* < 0.05, ** *p* < 0.01, *** *p* < 0.001.

**Table 10:** Summary of research questions’ answers.

Research Question (RQ)	Hypotheses	Associated Metrics	Type	Answer
**RQ1**: From a neurophysiological perspective, does the immersive AR on the packaging label of a product engage consumers differently compared to the static AR?	**H1a:** Immersive AR on packaging labels generates more emotional engagement compared to static AR.	EI	Neurophysiological	YES
	**H1b:** Immersive AR on packaging labels generate more cognitive engagement compared to static AR.	BATR	Neurophysiological	NO
**RQ2:** From a declarative perspective, does the immersive AR on the packaging label of a product change how the product is perceived compared to static AR?	**H2a:** Immersive AR on packaging labels generates more Perceived Informativeness (PI) compared to static AR.	PI	Declarative	NO
	**H2b:** Immersive AR on packaging labels generates more Perceived Brand Authenticity (PBA) compared to static AR.	PBA	Declarative	NO
	**H2c:** Immersive AR on packaging labels generates more Perceived Product Authenticity (PPA) compared to static AR.	PPA	Declarative	NO
	**H2d:** Immersive AR on packaging labels generates more Intention to Buy (ITB) compared to static AR.	ITB	Declarative	NO

## Data Availability

Data are available on request from the authors.

## Abbreviations

The following abbreviations are used in this manuscript:

AR	Augmented Reality
S	Static (AR)
I	Immersive (AR)
EI	Emotional Index
BATR	Beta/Alpha Theta Ratio
ARI	Augmented Reality Immersion questionnaire
PI	Perceived Informativeness
PBA	Perceived Brand Authenticity
PPA	Perceived Product Authenticity
ITB	Intention to Buy
